# The Application of Deep Brain Stimulation for Progressive Supranuclear Palsy: A Systematic Review

**DOI:** 10.3389/fneur.2022.827472

**Published:** 2022-06-02

**Authors:** Yafei Wen, Bin Jiao, Yafang Zhou

**Affiliations:** ^1^Department of Neurology, Xiangya Hospital, Central South University, Changsha, China; ^2^Department of Geriatrics Neurology, Xiangya Hospital, Central South University, Changsha, China

**Keywords:** progressive supranuclear palsy, deep brain stimulation, pedunculopontine nucleus, systematic review, Unified Parkinson's Disease Rating Scale (UPDRS)

## Abstract

Progressive supranuclear palsy (PSP) is a rare neurodegenerative disease, and currently no effective symptomatic or neuroprotective treatment is available for PSP. Deep brain stimulation (DBS), as a neurosurgical procedure, plays a role in a range of neurological and psychiatric disorders, and a series of case reports have applied DBS in PSP patients. However, there are no systematic investigations about the application of DBS in PSP patients; we therefore performed a systematic review to evaluate the efficacy of DBS for PSP. PubMed, EMBASE and the Cochrane library were systematically searched from database inception to July 31, 2021. Additionally, the reference lists of included studies were searched manually. Of 155 identified studies, 14 were eligible and were included in our analysis (*N* = 39 participants). We assessed the data between DBS-OFF and DBS-ON conditions, as measured by the Unified Parkinson's Disease Rating Scale (UPDRS) and other clinical rating scales. A reduction of UPDRS III scores under DBS-ON conditions in most PSP cases was observed, but the differences yielded no statistical significance. There was no sufficient evidence proving DBS was effective for PSP patients, though part of PSP cases could benefit from DBS and our findings could provide up-to-date information about the possible role of DBS in PSP, which would provide design strategies for following clinical trials and might ultimately help to promote the clinical application of DBS in PSP patients.

## Introduction

Progressive supranuclear palsy is the most common atypical parkinsonian disorder (1) with prominent four-repeat (4R-) tau neuropathology (2), and the classic phenotype termed Richardson's syndrome (PSP-RS, also known as Steele–Richardson–Olszewski syndrome) is characterized by prominent postural instability with repeated unprovoked falls, vertical supranuclear gaze palsy, akinetic-rigid parkinsonism with poor response to dopaminergic agents, and cognitive decline (3, 4). PSP is clinically heterogeneous, and several variant phenotypes have been gradually reported since PSP-RS was introduced in 1964 (3), including PSP-parkinsonism (PSP-P) (5), progressive gait freezing (PSP-PGF, ever referred to pure akinesia with gait freezing, PAGF) (6), and other 7 rare presentations (7, 8). PSP is a uniformly fatal disease with average disease duration of 8 years (9), and current medicine has limited efficacy in PSP (10). There are still no effective symptomatic or neuroprotective treatment available for PSP despite the transient benefit from levodopa therapy in the early stages of some cases (11).

As a neurosurgical procedure through implanting electrodes into specific targets within the brain and delivering electricity from an implanted battery source (12), deep brain stimulation (DBS) has become an important tool and has been applied to a range of neurological and psychiatric disorders mainly, including Parkinson's disease (PD), essential tremor, dystonia, epilepsy, and major depression (12, 13). PD is a common movement disorder, and muscular rigidity of limbs is an important clinical feature of PD (14), which is distinctive from PSP, the latter predominantly presenting with axial and gait symptoms. The subthalamic nucleus (STN) and globus pallidus interna (GPi) are common stimulating targets for treatments of PD in clinic, especially in cases without response to medication adjustments (15, 16). The pedunculopontine nucleus (PPN) is part of the mesencephalic locomotor region and plays a role in the initiation and maintenance of gait and balance (17). PPN has been proposed as a new target for DBS to treat movement disorders since the first PPN-DBS was carried out in a parkinsonian patient in 2005 (18). Studies have proven that patients with PD treated by PPN-DBS show improvements in gait disorder and falls (15, 19). Moreover, several researches have tried to apply PPN-DBS to treat patients with PSP and proposed PPN as a potential target for PSP (20–22).

The Unified Parkinson's Disease Rating Scale (UPDRS) (23), PSP rating score (PSPRS) (24) and freezing of gait questionnaire (FOG-Q) (25) are widely used clinical rating scales for parkinsonism, among which, UPDRS III and PSPRS are the most common objective assessments applied to reflect the effects of DBS on patients with PSP. Since there is still controversy over surgery benefits between different studies, herein, we carried out a study to evaluate the curative effects and provided a comprehensive summary of DBS for PSP.

## Methods

### Information Sources and Search Strategy

This systematic review has been organized according to the Preferred Reporting Items for Systematic Review and Meta-Analyses (PRISMA) statement guidelines (26) and has been registered at the International Prospective Register of Systematic Reviews (PROSPERO, registration number: CRD42020212628). We performed a comprehensive search of PubMed, EMBASE and the Cochrane library from database inception to July 31, 2021 using the following terms: “progressive supranuclear palsy” or “PSP” in association with “deep brain stimulation” or “DBS.” We scanned reference lists of relevant literature for additional potential sources. All publications were restricted to the English language, and all study designs were included.

### Study Selection and Data Extraction

Eligible literature had to meet all the inclusion criteria: (1) Subjects: PSP clinical diagnosis [NINDS-SPSP criteria in 1996 (4) and MDS-PSP criteria in 2017 (8) were considered for diagnosis]. (2) Interventions: any types of DBS. (3) Clinical assessments: outcome measures at baseline and follow-up; the UPDRS III is the primary outcome, and other clinical rating scales, including PSPRS, FOG-Q and GF-Q, are secondary outcomes. Reviews, animal research, repeated publications on patients and studies without complete data were excluded. Two independent investigators selected studies through reviewing the titles and abstracts in accordance with the inclusion and exclusion criteria. Disagreements between the two investigators were resolved by a third investigator.

Data were independently extracted by two investigators from each included study on (1) study information (including the first author, year of publication, country of centers); (2) patient characteristics (including age, gender, illness duration, and diagnostic criteria of PSP); (3) intervention (including surgical target for electrode implantation, proper voltage and frequency); (4) assessment of surgery effectiveness [including follow-up time, UPDRS part III scores (UPDRS III), PSPRS and other outcomes]. Additionally, we defined surgery effectiveness as improvement of the clinical rating scales by >30% to better show the surgical efficacy.

### Statistical Analysis

We divided the follow-up duration into two parts: short-term (<12 months after DBS) and long-term (≥12 months after DBS). We used the Wilcoxon rank sum test to compare the scores of UPDRS III under different conditions, for example, DBS-OFF vs DBS-ON, before surgery (baseline) vs after surgery (DBS-ON). PSPRS, FOG-Q, GF-Q and other outcomes could not be analyzed due to lack of enough data. Statistical analyses were performed using SPSS 25.0 for Windows, and *p* <0.05 was statistically significant.

## Result

### Description of Studies

**Figure 1:**
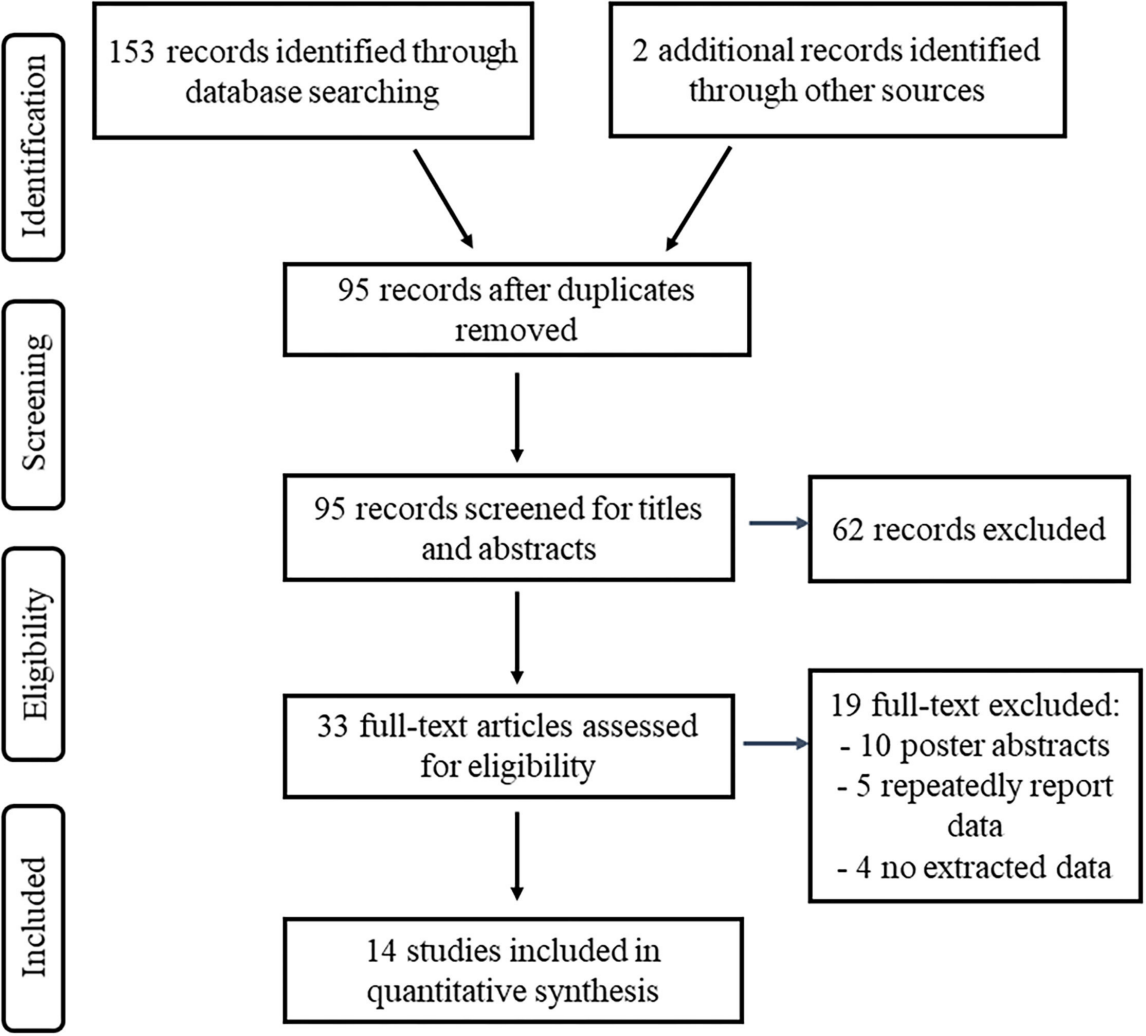
Flow of study information according to PRISMA statement, study selection, and reasons for exclusion.

A total of 155 articles of interest were searched and 95 articles identified after duplicates were removed (Figure 1). Of these, 62 articles were identified as irrelevant based on their titles and abstracts and were therefore eliminated. Among 33 potentially relevant articles, 10 were excluded because they were the abstracts of poster presentations; patients from five articles (27–31) overlapped with those in other studies (22, 32), and these five articles were excluded; four articles (33–36) had no extracted data and were excluded. A total of 14 articles were finally included in the analysis containing 39 patients with PSP comprising 19 patients with PSP-RS, 7 patients with PSP-P, 5 patients with PSP-PGF, and 8 patients without definite phenotypes. As for surgical targets, 35 patients were treated with PPN-DBS, 1 with STN-DBS, 1 with GPi-DBS, 2 with compound DBS. The basic characteristics of included studies are shown in Table 1.

**Table 1:** Baseline characteristics of the included studies.

**References**	**Sample size and gender**	**Mean age, ys**	**Duration, ys**	**Clinical diagnosis**	**DBS target**	**DBS parameters**			**Follow–up**	
						**Vol. V**	**Freq. Hz**	**Pulse width, us**	**Time, ms**	**Clinical evaluations**
Bergmann et al. (37)	1 F	55	8	PSP–P	bi–STN	L2.5; R3.5	185	60	9/42	UPDRS-III, cognitive tests and levodopa responsiveness
Brusa et al. (38)	1 M	70	3	PSP–P	uni PPN	3.4	25	NA	3/6/9	UPDRS-III, cognitive tests and FOG-Q
Lim et al. (39)	1 F; 1 M	59.5	NA	2 PSP	2 uni PPN	2–2.8	5–30	NA	7/10	Sleep stage distribution
Wilcox et al. (40)	1 M	69	8	PSP–PGF	bi PPN	L2.8–3.3 R3.5–3.8	35	60	2.5/5/7/10/15	FOG-Q and GF-Q
Ostrem et al. (41)	1 M	76	4	PSP–PGF	bi PPN	L4.5–5.1 R4.0–4.4	25	60	3/6/12	UPDRS and FOG-Q
Servello et al. (42)	3 M	68	NA	2 PSP–RS; 1 PSP–P	2 uni PPN; 1 uni PPN + bi GPi	NA	NA	NA	12/14	PSPRS-VI
Doshi et al. (20)	3 F; 1 M	60.8	3	2 PSP–RS; 2 PSP–P	4 bi PPN	0.7–3.5	20–45	60	6/18	PSPRS, UPDRS, PDQ-39 and adverse events
Oliveira Souza et al. (17)	1 F	74	NA	PSP–RS	bi PPN	2–4	20	60	1/3	UPDRS-III
Mazzone et al. (22)	4 NA	NA	NA	4 PSP	4 PPN	4.3–6.9	NA	60	0.5	UPDRS-III, Hoehn and Yahr
Scelzo et al. (32)	8 NA	NA	NA	8 PSP–RS	8 uni PPN	NA	NA	NA	6/12	PSPRS, UPDRS-III and adverse events
Galazky et al. (21)	5 F; 2 M	70	6.2	4 PSP–RS; 2 PSP–PGF; 1 PSP–P	6 bi PPN; 1 PPN + STN	3.5	8–130	60	3/12/24	UPDRS-III, TUG, PSP-QoL, cognitive tests and adverse events
Leimbach et al. (43)	1 F; 1 M	61	5	2 PSP	2 uni PPN	NA	NA	NA	12	Cognitive tests
Orcutt et al. (44)	1 M	75	4	PSP–RS	bi GPi	L 5.3; R 4.7	130	60	12	Improvement of AEO
Dayal et al. (45)	1 F; 2 M	66.7	8.7	1 PSP–RS; 1 PSP–P; 1 PSP–PGF	2 uni PPN; 1 bi–PPN	1.0–9.0	20–30	60	1/6/9/12	PSPRS, FOG-Q, GF-Q and adverse events

*DBS, deep brain stimulation; PSP, progressive supranuclear palsy; PSP-P, progressive supranuclear palsy-parkinsonism; PSP-RS, progressive supranuclear palsy-Richardson Syndrome; PPN, pedunculopontine nucleus; STN, subthalamic nucleus; GPi, globus pallidus internus; UPDRS, unified Parkinson's disease rating scale; PSPRS, progressive supranuclear palsy rating scale; FOG-Q, freezing of gait questionnaire; GF-Q, gait and falls questionnaire; PDQ-39, the 39-item Parkinson's disease questionnaire; TUG, timed up and go test; AEO, apraxia of eyelid opening; PSP-QoL, progressive supranuclear palsy quality of life scale*.

### The UPDRS III in PSP Patients

**Figure 2:**
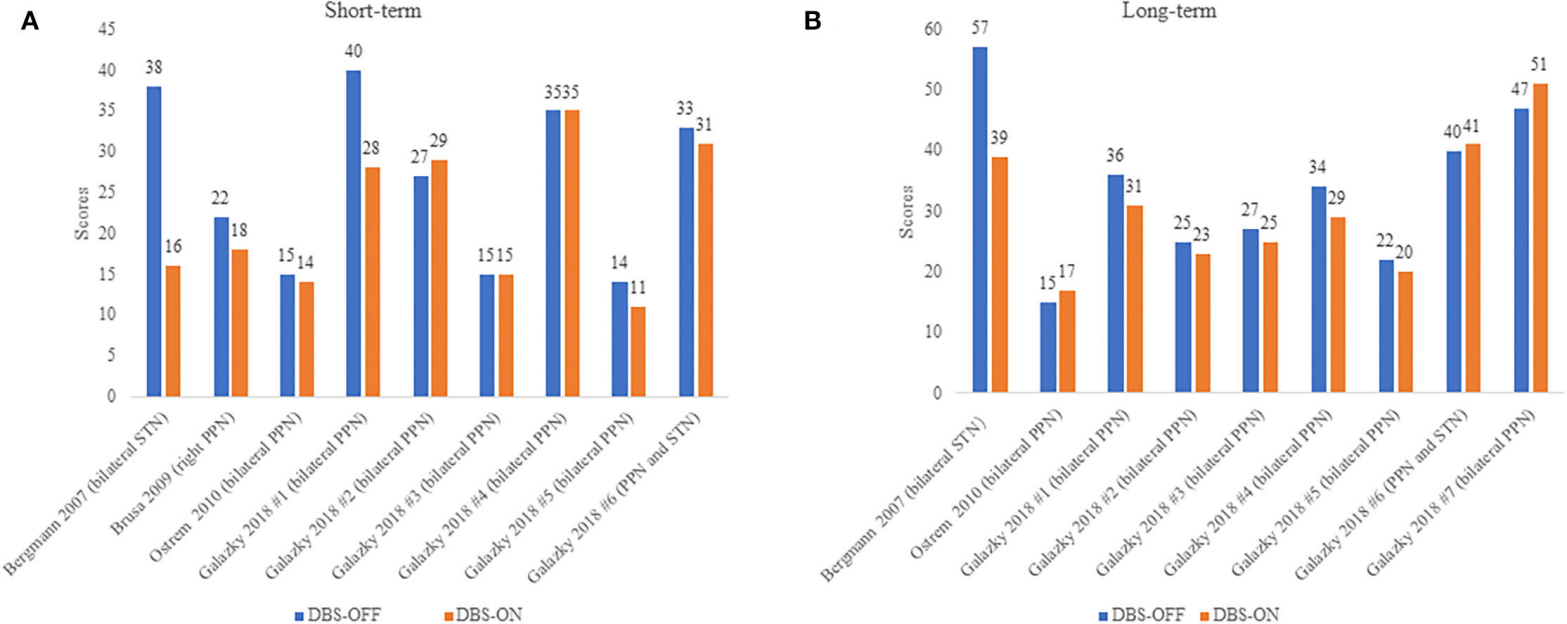
**(A,B)** UPDRS III scores in PSP patients under DBS-OFF and DBS-ON status. Short-term: <12 months after DBS, long-term: more than or equal to 12 months after DBS. The words in brackets are DBS targets of each patient.

Available data from four studies comprising 10 patients with PSP were included in this analysis comparing UPDRS III in patients with PSP between the DBS-OFF and DBS-ON status (Figure 2). Mazzone's (22) and Scelzo's studies (32) could not be analyzed since there were no detail scores in each patients with PSP. We divided the follow-up duration into two parts for subgroup analysis: short-term and long-term. In the short-term group, a total of nine PSP cases were analyzed (21, 37, 38, 41), and there was no statistically significant decrease in the UPDRS III scores under DBS-ON status though part of patients showed improvements (*p* = 0.051). Besides, the degree of amelioration was much smaller than those in Mazzone's study where the mean UPDRS III score in four patients with PSP lowered over 40% under DBS-ON conditions (22). In the long-term group, the data from nine patients with PSP were assessed (21, 37, 41) and the differences didn't reach the significance (*p* = 0.151), which was similar to the results of Scelzo's study where a total of eight patients with PSP-RS were treated by unilateral PPN-DBS and no obvious improvements were observed at 6 months or 12 months (32).

**Figure 3:**
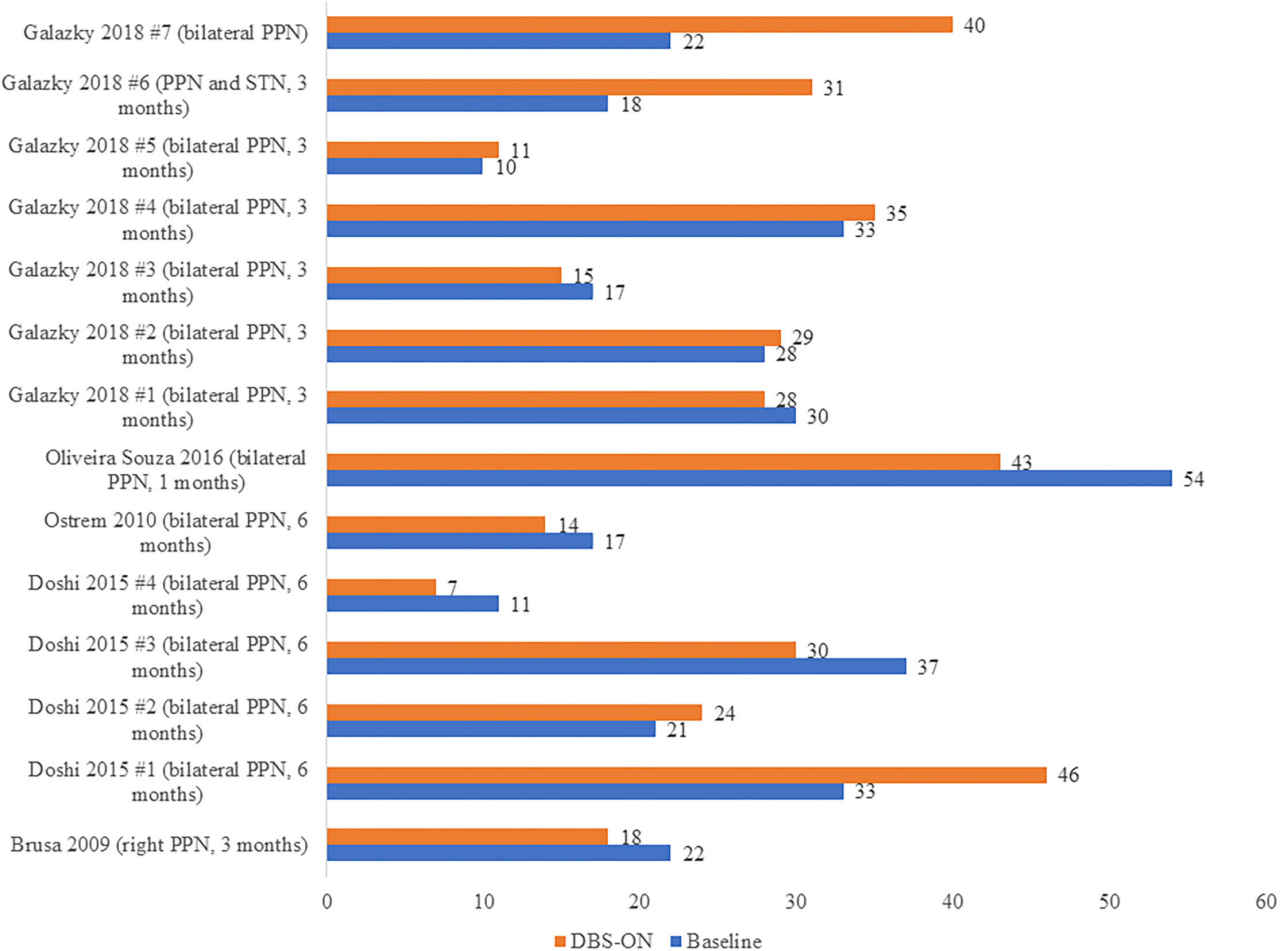
UPDRS III scores in PSP patients before DBS (Baseline) and after DBS (DBS-ON). The words in brackets are DBS targets and postoperative time point at which the “DBS-ON” assessment was performed in each patient.

On the other hand, we carried out another analysis using data from five articles (17, 20, 21, 38, 41) including 14 patients with PSP, and we compared the UPDRS III scores in these PSP patients pre-operation (at baseline) and post-operation (DBSON) as shown in Figure 3. If there were follow-up assessments at different time, we selected the date closest to the operation. No significant differences between these two groups were observed in the Wilcoxon rank sum test where we compared the score after operation to score at baseline (*p* = 1.000).

Considering different PSP clinical phenotypes might have different response to DBS surgery, we performed a simple analysis in short-term follow-up of patients presenting as PSP-RS, PSP-P, and PSP-PGF. As Table 2 shows, complete data were available in nine PSP cases from four studies (21, 37, 38, 41), and the mean improvement in PSP-P was higher than PSP-PS and PSP-PGF. We thus inferred different presentations of PSP might influence the efficacy of DBS, and patients with PSP-P might benefit more from DBS.

**Table 2:** UPDRS III scores of patients with different PSP phenotypes.

**Phenotypes**	**Surgery target**	**DBS-OFF (scores)**	**DBS-ON (scores)**	**Improvement**	**Mean**
PSP-RS	bilateral PPN-DBS (21)	40	28	30.0%	10.00%
	bilateral PPN-DBS (21)	35	35	0.0%	
	bilateral PPN-DBS (21)	15	15	0.0%	
PSP-PGF	bilateral PPN-DBS (21)	27	29	0.0%	9.37%
	bilateral PPN-DBS (21)	14	11	21.43%	
	bilateral PPN-DBS (41)	15	14	6.67%	
PSP-P	PPN- and STN-DBS (21)	33	31	6.06%	27.38%
	right PPN-DBS (38)	22	18	18.18%	
	bilateral STN-DBS (37)	38	16	57.89%	

### Unilateral vs Bilateral PPN-DBS for PSP Patients

A total of 35 PSP cases were treated through stimulating PPN alone; among these, 19 cases were assessed with clinical rating scales at baseline and follow-up and provided detailed information (17, 20, 21, 38, 40–42, 45). We divided these cases into two groups, unilateral PPN-DBS and bilateral PPN-DBS, and compared the improvements of short-term follow-up between these two groups. As Table 3 showed, the improvement of all five cases in the unilateral PPN group was <30%, while two cases (14.29%) in the bilateral PPN group reached the threshold of effectiveness, which to some degree indicated bilateral PPN stimulations might be more hopeful for PSP patients with mild symptoms than unilateral PPN stimulations. However, the overall surgery effectiveness of PPN-DBS in PSP patients was not very optimistic.

**Table 3:** The effectiveness of PPN-DBS for PSP patients.

**Stimulation**	**Clinical rating scales**	**Baseline (scores)**	**DBS-ON (scores)**	**Effectiveness**
Unilateral PPN (*N* = 5)	UPDRS III (38)	22	18	No
	PSPRS VI (42)	18	14	No
	PSPRS VI (42)	15	11	No
	PSPRS (45)	50	51	No
	PSPRS (45)	27	31	No
Bilateral PPN (*N* = 13)	FOG-Q (40)	16	7	Yes
	UPDRS III (41)	17	14	No
	PSPRS (45)	39	37	No
	UPDRS III (17)	54	43	No
	UPDRS III (21)	30	28	No
	UPDRS III (21)	28	29	No
	UPDRS III (21)	17	15	No
	UPDRS III (21)	33	35	No
	UPDRS III (21)	10	11	No
	UPDRS III (21)	22	40	No
	UPDRS III (20)	33	46	No
	UPDRS III (20)	21	24	No
	UPDRS III (20)	37	30	No
	UPDRS III (20)	11	7	Yes

*Surgery effectiveness was defined as improvement of the clinical rating scales by >30%*.

### Other Outcomes

Servello et al. followed up three PSP cases that underwent DBS and used PSPRS IV as the main outcome in the long term. They observed a reduction in the number of falls and an amelioration of postural balance in all patients, which was an encouraging result (42). Another three studies also evaluated PSPRS in their cases and reported that there was no obvious improvement (20, 32, 45). In total, four cases from four studies provided available FOG-Q scores: two patients with PSP-P (38, 45) and two PSP-PGF patients (40, 41). The FOG-Q scores among these cases averagely reduced 33.8% at the short-term follow-up visit, with a reduction of more than 50% in a patient with PSP-PGF and a patients with PSP-P. However, the sample size was too small to perform statistical analysis.

One article observed a great improvement of apraxia of eyelid opening (AEO) in a patients with PSP through bilateral GPi stimulations (44). Lim and collaborators proved that PPN-DBS significantly increased nocturnal rapid eye movement sleep in five cases including two patients with PSP (39), which linked PPN with sleep and extended the functions of PPN-DBS. Leimbach et al. focused on the effects of PPN-DBS on cognition through evaluating a comprehensive battery of neuropsychological assessment in five PD cases and two PSP cases. They concluded that PPN-DBS was generally safe from a cognitive perspective though there was no significant change after surgery (43), which was consistent with the results from other studies on cognitive domains (37, 38).

Additionally, four studies mentioned the adverse events related to DBS. Intraoperative bleeding is a major surgical complication worthy strong attention, and it occurred in two patients in PPN-DBS with unknown reasons in Scelzo's cohort where chronic stimulation itself was well tolerated (32), which indicated intracranial hemorrhage during surgery should be better investigated in further studies especially considering the possibility of underreporting due to a negative publication bias. Other surgical adverse events included apathy and a buccofacial apraxia, which were transient and recoverable (21). As for stimulation-related adverse events, paresthesia, oscillopsia, diplopia and dysarthria were observed (20, 21, 45).

## Discussion

The aim of this study was to summarize the efficacy of DBS in patients with PSP through analyzing related articles. To our knowledge, this is the first systematic review of DBS for PSP even though we were only able to combine results from 14 studies. In most cases, the clinical rating scales ameliorated under DBS-ON conditions compared to those under DBS-OFF conditions; however, we found no statistical significances. Additional analyses indicated that the durations of follow-up time, phenotypes of PSP and unilateral or bilateral PPN-DBS might influence the degree of clinical scales improvements. We further found DBS is associated with sleep, AEO and cognitive functions of PSP patients in addition to axial symptoms like falls and gait disorders.

The treatment of PSP is changing since currently, no effective symptomatic or neuroprotective treatment is available for PSP (10), and several clinical trials showed no beneficial effects in PSP patients (46). DBS is a potentially promising tool to provide symptomatic benefit for PSP. Galazky et al. proposed that bilateral PPN-DBS resulted in frequency-dependent effects in PSP patients and they observed low frequency improved cyclic gait parameters while high frequency ameliorated hypokinesia (21), which indicates that choosing proper stimulator parameters for individualized patients is essential. About one PSP case treated by double implanted GPi-PPN gained a better clinical outcome (42). Considering that basal ganglia and brainstem are generally affected in PSP patients (47), there may be an increased synergic effect existing when simultaneously stimulating different nucleus if the patient is tolerant.

PPN is a new target of DBS, and several studies have supported the positive effects of PPN-DBS for PD (15, 19, 48). Garcia-Rill et al. concluded some possible mechanisms of how stimulation in the PPN area could improve gait (49), which mainly results from the complex anatomy and multiple projections of PPN. Pathological study observed that cholinergic and noncholinergic neuronal populations in the PPN were significantly reduced in PSP patients, and this discovery suggested an underlying pathological physiological link could exist between PSP and PPN cell loss (50), which provided evidence for the application of PPN-DBS for PSP patients. Target section within the PPN region could lead to the variability of clinical response (45, 51), which to some extent, can explain why different studies showed variable outcomes. In addition, the variability may be also partly attributable to variations in stimulation parameters, unilateral versus bilateral stimulation, isolated PPN stimulation versus combining the PPN with other targets, duration of follow-up, disease severity and progression, outcome measures used, as well as different PSP phenotypes (45). Therefore, in order to optimize the curative effect of PPN-DBS for PSP, it is important to further understand the anatomy of PPN, improve the localization of the optimal targets and design appropriate parameters.

PSP-P shows a better response to levodopa medications and a more favorable course with longer survival than PSP-RS (52). The present review found PSP-P patients also presented a higher improvement after DBS surgery compared with PSP-RS and PSP-PGF patients, which might result from the various disease severity and different response to levodopa in patients with different phenotypes. On the other hand, we observed that the levodopa equivalent daily dose was largely reduced in a PSP-P patient in Bergmann's study (37), while part of patients did not reduce levodopa equivalent daily dose after DBS surgery (21), which indicated the effects that the DBS surgery might make on levodopa dose in PSP patients needed further explorations. However, there was not adequate information about levodopa response in included patients, which also restricted the discussion about the interactions between DBS surgery and levodopa response in PSP patients to whether a better response to levodopa leads to better response to DBS and whether DBS surgery changes the response to levodopa in PSP patients.

Cognitive decline is a common clinical symptom in PSP patients, and fronto-executive deficits are the dominated neuropsychological profile of PSP (53). Compared to other parkinsonian syndromes, cognitive progression is more severe and rapid in PSP (53). DBS is generally safe for cognitive function in PD patients (43, 54, 55), and STN-DBS even can improve cognitive function to a certain extent in PD (55). However, there are only a few studies that have investigated the effects of DBS on cognitive condition in PSP patients, and the sample is small and heterogeneous (37, 38, 43). Current evidence indicates PPN-DBS might be safe for PSP patients from a cognitive perspective (38, 43), and more studies are needed to explore the associations between DBS and cognitive function in PSP patients.

This review has several limitations. The major limitation is the relatively small number of included studies as well as the small number of eligible participants. Second, some of included studies are case reports and the data from several studies are incomplete or unavailable, which gains the bias of statistical outcomes and another main limitation for the studies used in this review is possible selection bias: considering PSP could show aggressive progression, relatively benign and early-stage patients might be the candidate for DBS. Moreover, it is an important limitation to analyze the clinical scales, which were performed in different cases where there were no consistent stimulation procedures, DBS parameters, and washout periods. Finally, the outcome of our study is simple: though UPDRS III as the primary outcome was well analyzed, we really desire more motor and non-motor scales to evaluate the DBS for PSP, especially disease-specific outcomes like PSPRS, and the safety of DBS in PSP patients still needs more discussions since only some studies reported adverse events. Thus, more well-designed research with larger cohorts is well needed.

## Conclusion

This review investigated the application of DBS in PSP patients, however there was not sufficient evidence proving DBS was effective for PSP patients though part of PSP cases could benefit from DBS. Our findings gave up-to-date information about the possible role of DBS in PSP, which would provide design strategies for following clinical trials and ultimately help improve the clinical application of DBS in PSP patients.

## Data Availability Statement

The original contributions presented in the study are included in the article/supplementary material, further inquiries can be directed to the corresponding author.

## Author Contributions

YW searched the articles, selected, and assessed the articles, extracted, analyzed the data, and drafted the manuscript. BJ made contributions to articles selection, data analysis, and manuscript revision. YZ contributed to study design, acquisition of data, assessment of articles, analysis, and interpretation of data, drafting and revising the manuscript, and as well as supporting this study. All authors contributed to the article and approved the submitted version.

## Funding

This study was supported the National Key R&D Program of China (Nos. 2020YFC2008500 and 2018YFC1312003), the National Natural Science Foundation of China (Nos. 81671075, 81971029, 82071216, and 81901171), Hunan Innovative Province Construction Project (No. 2019SK2335), and the Youth Science Foundation of Xiangya Hospital (No. 2018Q020).

## Conflict of Interest

The authors declare that the research was conducted in the absence of any commercial or financial relationships that could be construed as a potential conflict of interest.

## Publisher's Note

All claims expressed in this article are solely those of the authors and do not necessarily represent those of their affiliated organizations, or those of the publisher, the editors and the reviewers. Any product that may be evaluated in this article, or claim that may be made by its manufacturer, is not guaranteed or endorsed by the publisher.

## References

[1] Olfati N Shoeibi A Litvan I. Progress in the treatment of Parkinson-Plus syndromes. Parkinsonism Relat Disord. (2019) 59:101-10. 10.1016/j.parkreldis.2018.10.006 30314846

[2] Rosler TW Tayaranian Marvian A Brendel M Nykanen NP Hollerhage M Schwarz SC . Four-repeat tauopathies. Prog Neurobiol. (2019) 180:101644. 10.1016/j.pneurobio.2019.101644 31238088

[3] Steele JC Richardson JC Olszewski J. Progressive supranuclear palsy a heterogeneous degeneration involving the brain stem, basal ganglia and cerebellum with vertical gaze and pseudobulbar palsy, nuchal dystonia and dementia. Arch Neurol. (1964) 10:333-59. 10.1001/archneur.1964.00460160003001 14107684

[4] Litvan I Agid Y Calne D Campbell G Dubois B Duvoisin RC . Clinical research criteria for the diagnosis of progressive supranuclear palsy (Steele-Richardson-Olszewski syndrome): report of the NINDS-SPSP international workshop. Neurology. (1996) 47:1-9 . 10.1212/WNL.47.1.1 8710059

[5] Williams DR Lees AJ. Progressive supranuclear palsy: clinicopathological concepts and diagnostic challenges. Lancet Neurol. (2009) 8:270-9. 10.1016/S1474-4422(09)70042-0 19233037

[6] Owens E Josephs KA Savica R Hassan A Klassen B Bower J . The clinical spectrum and natural history of pure akinesia with gait freezing. J Neurol. (2016) 263:2419-23. 10.1007/s00415-016-8278-x 27624121

[7] Ling H. Clinical approach to progressive supranuclear palsy. J Mov Disord. (2016) 9:3-13. 10.14802/jmd.15060 26828211 PMC4734991

[8] Höglinger GU Respondek G Stamelou M Kurz C Josephs KA Lang AE . Clinical diagnosis of progressive supranuclear palsy: the movement disorder society criteria. Mov Disord. (2017) 32:853-64. 10.1002/mds.26987 28467028 PMC5516529

[9] Levin J Kurz A Arzberger T Giese A Hoglinger GU. The differential diagnosis and treatment of atypical Parkinsonism. Dtsch Arztebl Int. (2016) 113:61-9. 10.3238/arztebl.2016.0061 26900156 PMC4782269

[10] Koros C Stamelou M. Interventions in progressive supranuclear palsy. Parkinsonism Relat Disord. (2016) 22(Suppl 1):S93-95. 10.1016/j.parkreldis.2015.09.033 26459661

[11] Boxer AL Yu JT Golbe LI Litvan I Lang AE Höglinger GU. Advances in progressive supranuclear palsy: new diagnostic criteria, biomarkers, and therapeutic approaches. Lancet Neurol. (2017) 16:552-63. 10.1016/S1474-4422(17)30157-6 28653647 PMC5802400

[12] Lozano AM Lipsman N Bergman H Brown P Chabardes S Chang JW . Deep brain stimulation: current challenges and future directions. Nat Rev Neurol. (2019) 15:148-60. 10.1038/s41582-018-0128-2 30683913 PMC6397644

[13] Ramirez-Zamora A Giordano J Boyden ES Gradinaru V Gunduz A Starr PA . proceedings of the sixth deep brain stimulation think tank modulation of brain networks and application of advanced neuroimaging, neurophysiology, and optogenetics. Front Neurosci. (2019) 13:936. 10.3389/fnins.2019.00936 31572109 PMC6751331

[14] Kalia LV Lang AE. Parkinson's disease. The Lancet. (2015) 386:896-912. 10.1016/S0140-6736(14)61393-3 25904081

[15] Lin F Wu D Lin C Cai H Chen L Cai G . Pedunculopontine nucleus deep brain stimulation improves gait disorder in parkinson's disease: a systematic review and meta-analysis. Neurochem Res. (2020) 45:709-19. 10.1007/s11064-020-02962-y 31950450

[16] Armstrong MJ Okun MS. Diagnosis and treatment of parkinson disease: a review. JAMA. (2020) 323:548-60. 10.1001/jama.2019.22360 32044947

[17] de Oliveira Souza C de Lima-Pardini AC Coelho DB Brant Machado R Alho EJL Di Lorenzo Alho AT . Peduncolopontine DBS improves balance in progressive supranuclear palsy: instrumental analysis. Clin Neurophysiol. (2016) 127:3470-1. 10.1016/j.clinph.2016.09.006 27721105

[18] Mazzone P Scarnati E Garcia-Rill E. Commentary: the pedunculopontine nucleus: clinical experience, basic questions and future directions. J Neural Transm (Vienna). (2011) 118:1391-6. 10.1007/s00702-010-0530-4 21188437 PMC3654381

[19] Thevathasan W Debu B Aziz T Bloem BR Blahak C Butson C . Pedunculopontine nucleus deep brain stimulation in Parkinson's disease: a clinical review. Mov Disord. (2018) 33:10-20. 10.1002/mds.27098 28960543

[20] Doshi PK Desai JD Karkera B Wadia PM. Bilateral pedunculopontine nucleus stimulation for progressive supranuclear palsy. Stereotact Funct Neurosurg. (2015) 93:59-65. 10.1159/000368702 25662728

[21] Galazky I Kaufmann J Lorenzl S Ebersbach G Gandor F Zaehle T . Deep brain stimulation of the pedunculopontine nucleus for treatment of gait and balance disorder in progressive supranuclear palsy: Effects of frequency modulations and clinical outcome. Parkinsonism Relat Disord. (2018) 50:81-6. 10.1016/j.parkreldis.2018.02.027 29503154

[22] Mazzone P Vilela Filho O Viselli F Insola A Sposato S Vitale F . Our first decade of experience in deep brain stimulation of the brainstem: elucidating the mechanism of action of stimulation of the ventrolateral pontine tegmentum. J Neural Transm (Vienna). (2016) 123:751-67. 10.1007/s00702-016-1518-5 26865208

[23] Disease MDSTFoRSfPs. The Unified Parkinson's Disease Rating Scale (UPDRS): status and recommendations. Mov Disord. (2003) 18, 738-50. 10.1002/mds.10473 12815652

[24] Golbe LI Ohman-Strickland PA. A clinical rating scale for progressive supranuclear palsy. Brain. (2007) 130:1552-65. 10.1093/brain/awm032 17405767

[25] Giladi Shabtai Simon Biran Tal Korczyn. Construction of freezing of gait questionnaire for patients with Parkinsonism. Parkinsonism Relat Disord. (2000) 6:165-70. 10.1016/S1353-8020(99)00062-0 10817956

[26] Moher D Liberati A Tetzlaff J Altman DG Group P. Preferred reporting items for systematic reviews and meta-analyses: the PRISMA statement. BMJ. (2009) 339:b2535. 10.1136/bmj.b2535 19622551 PMC2714657

[27] Hazrati LN Wong JC Hamani C Lozano AM Poon YY Dostrovsky JO . Clinicopathological study in progressive supranuclear palsy with pedunculopontine stimulation. Mov Disord. (2012) 27:1304-7. 10.1002/mds.25123 22865554

[28] Galazky I Kaufmann J Voges J Hinrichs H Heinze HJ Sweeney-Reed CM. Neuronal spiking in the pedunculopontine nucleus in progressive supranuclear palsy and in idiopathic Parkinson's disease. J Neurol. (2019) 266:2244-51. 10.1007/s00415-019-09396-9 31155683

[29] Mazzone P Insola A Sposato S Scarnati E. The deep brain stimulation of the pedunculopontine tegmental nucleus. Neuromodulation. (2009) 12:191-204 10.1111/j.1525-1403.2009.00214.x 22151360

[30] Mazzone P Sposato S Insola A Scarnati E. The clinical effects of deep brain stimulation of the pedunculopontine tegmental nucleus in movement disorders may not be related to the anatomical target, leads location, and setup of electrical stimulation. Neurosurgery. (2013) 73:894-906. 10.1227/NEU.0000000000000108 23867299

[31] Galazky I Zaehle T Sweeney-Reed CM Neumann J Heinze HJ Voges J . Neuronal oscillations of the pedunculopontine nucleus in progressive supranuclear palsy: influence of levodopa and movement. Clin Neurophysiol. (2020) 131:414-9. 10.1016/j.clinph.2019.11.033 31877491

[32] Scelzo E Lozano AM Hamani C Poon YY Aldakheel A Zadikoff C . Peduncolopontine nucleus stimulation in progressive supranuclear palsy: a randomised trial. J Neurol Neurosurg Psychiatry. (2017) 88:613-6. 10.1136/jnnp-2016-315192 28214797

[33] Sun L Hinrichs H. Moving average template subtraction to remove stimulation artefacts in EEGs and LFPs recorded during deep brain stimulation. J Neurosci Methods. (2016) 266:126-136. 10.1016/j.jneumeth.2016.03.020 27039973

[34] Weinberger M Hamani C Hutchison WD Moro E Lozano AM Dostrovsky JO. Pedunculopontine nucleus microelectrode recordings in movement disorder patients. Exp Brain Res. (2008) 188:165-74. 10.1007/s00221-008-1349-1 18347783

[35] Yeh IJ Tsang EW Hamani C Moro E Mazzella F Poon YY . Somatosensory evoked potentials recorded from the human pedunculopontine nucleus region. Mov Disor. (2010) 25:2076-83. 10.1002/mds.23233 20669321

[36] Tattersall TL Stratton PG Coyne TJ Cook R Silberstein P Silburn PA . Imagined gait modulates neuronal network dynamics in the human pedunculopontine nucleus. Nat Neurosci. (2014) 17:449-54. 10.1038/nn.3642 24487235

[37] Bergmann KJ Salak VL. Subthalamic stimulation improves levodopa responsive symptoms in a case of progressive supranuclear palsy. Parkinsonism Relat Disord. (2008) 14:348-52. 10.1016/j.parkreldis.2007.07.004 17825599

[38] Brusa L Iani C Ceravolo R Galati S Moschella V Marzetti F . Implantation of the nucleus tegmenti pedunculopontini in a PSP-P patient: safe procedure, modest benefits. Mov Disord. (2009) 24:2020-2. 10.1002/mds.22706 19672983

[39] Lim AS Moro E Lozano AM Hamani C Dostrovsky JO Hutchison WD et al. Selective enhancement of rapid eye movement sleep by deep brain stimulation of the human pons. Ann Neurol. (2009) 66:110-4. 10.1002/ana.21631 19670451

[40] Wilcox RA Cole MH Wong D Coyne T Silburn P Kerr G. Pedunculopontine nucleus deep brain stimulation produces sustained improvement in primary progressive freezing of gait. J Neurol Neurosurg Psychiatry. (2010) 82:1256-9. 10.1136/jnnp.2010.213462 20971757

[41] Ostrem JL Christine CW Glass GA Schrock LE Starr PA. Pedunculopontine nucleus deep brain stimulation in a patient with primary progressive freezing gait disorder. Stereotact Funct Neurosurg. (2010) 88:51-5. 10.1159/000268742 20051710

[42] Servello D Zekaj E Saleh C Menghetti C Porta M. Long-term follow-up of deep brain stimulation of peduncolopontine nucleus in progressive supranuclear palsy: report of three cases. Surg Neurol Int. (2014) 5:S416-420. 10.4103/2152-7806.140208 25289173 PMC4173638

[43] Leimbach F Gratwicke J Foltynie T Limousin P Zrinzo L Jahanshahi M. The effects of deep brain stimulation of the pedunculopontine nucleus on cognition in Parkinson's disease and progressive supranuclear palsy. Clin Park Relat Disord. (2019) 1:48-51. 10.1016/j.prdoa.2019.08.001 34316599 PMC8288563

[44] Orcutt T Vitek J Patriat R Harel N Matsumoto J. Apraxia of eyelid opening improved by pallidal stimulation in progressive supranuclear palsy. Mov Disord Clin Pract. (2020) 7:698-700. 10.1002/mdc3.13001 32775519 PMC7396836

[45] Dayal V Rajabian A Jahanshahi M Aviles-Olmos I Cowie D Peters A . Pedunculopontine nucleus deep brain stimulation for parkinsonian disorders: a case series. Stereotact Funct Neurosurg. (2020) 1-8. 10.1159/000511978 33279909

[46] Coughlin DG Litvan I. Progressive supranuclear palsy: advances in diagnosis and management. Parkinsonism Relat Disord. (2020) 73:105-16. 10.1016/j.parkreldis.2020.04.014 32487421 PMC7462164

[47] Dickson DW Rademakers R Hutton ML. Progressive supranuclear palsy: pathology and genetics. Brain Pathol. (2007) 17:74-82. 10.1111/j.1750-3639.2007.00054.x 17493041 PMC8095545

[48] Yousif N Bhatt H Bain PG Nandi D Seemungal Seemungal BM The effect of pedunculopontine nucleus deep brain stimulation on postural sway and vestibular perception. Eur J Neurol. (2016) 23:668-70. 10.1111/ene.12947 26800658 PMC4819708

[49] Garcia-Rill E Saper CB Rye DB Kofler M Nonnekes J Lozano A . Focus on the pedunculopontine nucleus. Consensus review from the May 2018 brainstem society meeting in Washington, DC, USA. Clin Neurophysiol. (2019) 130:925-40. 10.1016/j.clinph.2019.03.008 30981899 PMC7365492

[50] Sébille SB Rolland AS Faillot M Perez-Garcia F Colomb-Clerc A Lau B . Normal and pathological neuronal distribution of the human mesencephalic locomotor region. Mov Disord. (2019) 34:218-27. 10.1002/mds.27578 30485555

[51] Goetz L Bhattacharjee M Ferraye MU Fraix V Maineri C Nosko D . deep brain stimulation of the pedunculopontine nucleus area in parkinson disease: mri-based anatomoclinical correlations and optimal target. Neurosurgery. (2019) 84:506-18. 10.1093/neuros/nyy151 29846707

[52] Jecmenica-Lukic M Petrovic IN Pekmezovic T Kostic VS. Clinical outcomes of two main variants of progressive supranuclear palsy and multiple system atrophy: a prospective natural history study. J Neurol. (2014) 261:1575-83. 10.1007/s00415-014-7384-x 24888315

[53] Fiorenzato E Antonini A Camparini V Weis L Semenza C Biundo Biundo R Characteristics and progression of cognitive deficits in progressive supranuclear palsy vs. multiple system atrophy and Parkinson's disease. J Neural Transm (Vienna). (2019) 126:1437-45. 10.1007/s00702-019-02065-1 31432258

[54] Philipson J Blomstedt P Fredricks A Hariz M Stenmark Persson R Jahanshahi M. Short- and long-term cognitive effects of deep brain stimulation in the caudal zona incerta versus best medical treatment in patients with Parkinson's disease. J Neurosurg. (2020) 7:1-9. 10.3171/2019.12.JNS192654 32032954

[55] You Z Wu YY Wu R Xu ZX Wu X Wang Wang XP Efforts of subthalamic nucleus deep brain stimulation on cognitive spectrum: from explicit to implicit changes in the patients with Parkinson's disease for 1 year. CNS Neurosci Ther. (2020) 26:972-80. 10.1111/cns.13392 32436660 PMC7415202

